# Comprehensive Molecular Sampling Yields a Robust Phylogeny for Geometrid Moths (Lepidoptera: Geometridae)

**DOI:** 10.1371/journal.pone.0020356

**Published:** 2011-06-01

**Authors:** Pasi Sihvonen, Marko Mutanen, Lauri Kaila, Gunnar Brehm, Axel Hausmann, Hermann S. Staude

**Affiliations:** 1 Research Funding Services, University of Helsinki, Helsinki, Finland; 2 Zoological Museum, University of Oulu, Oulu, Finland; 3 Finnish Museum of Natural History, University of Helsinki, Helsinki, Finland; 4 Institut für Spezielle Zoologie und Evolutionsbiologie mit Phyletischem Museum, Jena, Germany; 5 Staatliche Naturwissenschaftliche Sammlungen Bayerns, München, Germany; 6 Magaliesburg, South Africa; Biodiversity Insitute of Ontario - University of Guelph, Canada

## Abstract

**Background:**

The moth family Geometridae (inchworms or loopers), with approximately 23 000 described species, is the second most diverse family of the Lepidoptera. Apart from a few recent attempts based on morphology and molecular studies, the phylogeny of these moths has remained largely uninvestigated.

**Methodology/Principal Findings:**

We performed a rigorous and extensive molecular analysis of eight genes to examine the geometrid affinities in a global context, including a search for its potential sister-taxa. Our maximum likelihood analyses included 164 taxa distributed worldwide, of which 150 belong to the Geometridae. The selected taxa represent all previously recognized subfamilies and nearly 90% of recognized tribes, and originate from all over world. We found the Geometridae to be monophyletic with the Sematuridae+Epicopeiidae clade potentially being its sister-taxon. We found all previously recognized subfamilies to be monophyletic, with a few taxa misplaced, except the Oenochrominae+Desmobathrinae complex that is a polyphyletic assemblage of taxa and the Orthostixinae, which was positioned within the Ennominae. The Sterrhinae and Larentiinae were found to be sister to the remaining taxa, followed by Archiearinae, the polyphyletic assemblage of Oenochrominae+Desmobathrinae moths, Geometrinae and Ennominae.

**Conclusions/Significance:**

Our study provides the first comprehensive phylogeny of the Geometridae in a global context. Our results generally agree with the other, more restricted studies, suggesting that the general phylogenetic patterns of the Geometridae are now well-established. Generally the subfamilies, many tribes, and assemblages of tribes were well supported but their interrelationships were often weakly supported by our data. The Eumeleini were particularly difficult to place in the current system, and several tribes were found to be para- or polyphyletic.

## Introduction

The family Geometridae (inchworms or loopers), is one of the two most diverse families of Lepidoptera, with approximately 23 000 described species [1]–[3], occurring worldwide except in the polar regions. In larvae of Geometridae the ventral prolegs of segments A3–A5 are usually absent or vestigial, causing the typical looping movement. The adult Geometridae are generally rather slender-bodied, broad-winged, and somewhat delicate, but several robust-built lineages exist. The majority of the species are nocturnal and cryptically patterned, but several lineages include brightly-coloured diurnal species. Several species are defoliators of some economic importance [4]. The vast majority of geometrid larvae are external feeders, mainly on leaves, but certain lineages specialize on flowers and developing seeds and fruit. In Hawaii, an endemic radiation of *Eupithecia* Curtis (Larentiinae) has predatory larvae [5].

Morphologically the geometrids are best defined by the unique structure of the tympanal organs, particularly the presence of the ansa, found at the base of the abdomen and have their tympanal apertures opening ventro-laterally. These structures are reduced or lost in some of the brachypterous females [6].

The alpha-taxonomy of the Geometridae has been developing progressively, and excellent treatises exist, but these are often geographically limited and not aimed at resolving geometrid phylogeny at a deeper global level. Our current knowledge of phylogenetic relationships is largely based on Holloway's [7]–[10] morphological works on the Bornean fauna, where the findings were placed in a wider taxonomic concept. Other recent significant contributions, which treat more restricted taxa, include for instance works on the Neotropical Ennominae [11], the Macariini [12], the Sterrhinae [13], the Scopulini [14], *The Geometrid Moths of Europe* series [15]–[17] and *The Moths of North America* [18], [19]. In recent years these morphological findings have been tested and supported by DNA studies [20]–[24]. Forum Herbulot [25], which is an international scientific community with research focused on Geometridae, has attempted to create a synthesis of all available information. According to Forum Herbulot [25], currently eight subfamilies are recognized in the following tentative order with species' numbers from Scoble & Hausmann [2]: Sterrhinae (2940), Larentiinae (6228), Geometrinae (2529), Archiearinae (18), Oenochrominae (328), Desmobathrinae (248), Orthostixinae (17) and Ennominae (10 682). These are divided further into 85 tribes in current use.

Previous DNA analyses have suffered from two major limitations: firstly a lack of comprehensive taxon sampling, both taxonomically and geographically, and secondly only a limited number of phylogenetically informative genetic markers have been analysed.

The main objective of this research has been to provide a solid evolutionary framework for the described Geometridae, aimed at clarifying broad patterns at three levels: the relationship between the Geometridae and potential sister-taxa, the relationship between the larger clades (subfamilies) within the family, and the relationship between subordinated taxa (tribes and genera). We hope that the synthetic approach will provide a solid basis for further studies, whether taxonomic or applied.

## Methods

### Taxon sampling and specimen acquisition

Most specimens analysed were gathered from the DNA sample collections of the authors. In cases where DNA samples preserved in ethanol were not available, we extracted DNA from dry collection samples less than 15 years old. Overall, DNA extracted from ethanol preserved samples was of a high quality, while DNA extracted from dry samples was generally of lower quality, and many taxa had to be excluded due to limited sequencing success. Additional taxon samples were received from several collectors (*vide* Acknowledgments) and from the AtoL/LepTree DNA collection at the University of Maryland (http://www.leptree.net). Published sequences (created by Niklas Wahlberg) of three taxa were also included.

We sampled the described geometrid diversity at the tribal level as comprehensively as possible, using a summary of the classification of the Geometridae by Forum Herbulot [25] as our working hypothesis. This classification is largely based on revisions by Holloway [7]–[10]. We also supplemented the taxon coverage by including 31 important taxa of doubtful affinity, e.g. [22], [24], [26]. Two or more examples were included for several tribes, especially in the tribes distributed widely such as the Nacophorini (Ennominae). Representative taxa from all eight recently recognized geometrid subfamilies were represented and material from 76/85 recently recognized tribes (89.4% of all) were obtained. In addition 31 taxa currently not assigned to tribes were also included. A total of 164 taxa were analysed, 150 being members of the Geometridae (Table S1). Specimens were sampled from the following regions: 69 from the Palaearctic Region, 37 from the Neotropical Region, 13 from the Afrotropical Region, 10 from Southeast Asia, 13 from Australia and 8 from New Zealand. Specimens from the Nearctic Region were not sampled because some Holarctic genera were included and the Nearctic taxa are classified into higher categories that were already represented in the analysis by the Palaearctic and the Neotropical material.

To reduce the risk of misidentification, all the specimens were cross-checked with their DNA barcodes in BOLD (Barcode of Life Data Systems, http://www.barcodinglife.org/views/login.php) [27], where reference specimens were available for more than 10 000 geometrid species including most of the species used in this study. Identification of the many Neotropical taxa are preliminary because for many groupings there are is no current identification information available, see for instance [28]. Material was compared to relevant type material, but in many instances the comparisons were based on wing patterns only.

Taxonomic data for sequenced taxa, sample ID, collection information, current systematic placement, and references to relevant literature where the tribal association is used, are shown in Table S1. For a full overview, it also includes nine tribes not covered by our study. GenBank accession numbers and sequencing success are provided in Table S2.

Our research did not specifically attempt to resolve affinities among non-geometrid taxa (Sematuridae and Uraniidae), but we included all such obtained material as either one of them was likely to represent a possible sister-group to Geometridae [29]. We also included members of Cimeliidae, Epicopeiidae and Drepanidae as their affinity to Geometroidea has remained doubtful. Of these, Epicopeiidae (currently a family in Drepanoidea) may actually be more closely related to Geometroidea than Drepanoidea [29].

### Molecular techniques

Usually legs, but sometimes larger body parts of adult specimens were used for DNA extraction. The remaining parts of specimens were preserved as dry samples to serve as vouchers. Body parts to be used in DNA extraction were dried and powdered, and DNA was extracted and purified using Qiagen's DNeasy™ extraction kit following manufacturer's instructions.

Regions from one mitochondrial gene and seven nuclear genes were combined to form a data matrix. We sequenced altogether 1476 base pairs from *cytochrome oxidase subunit 1 gene* (CO1) of the mitochondrial genome, and altogether 4681 base pairs from *Elongation Factor-1α* (EF-1α), *Ribosomal protein S5* (RpS5), *Carbamoylphosphate synthase domain protein* (CAD), *Cytosolic malate dehydrogenase* (MDH), *Glyceraldehyde-3-phosphate dehydrogenase* (GAPDH), *Isocitrate dehydrogenase* (IDH) and *wingless* genes of the nuclear genome. The data accounted for a total of 6157 base pairs. DNA amplification and sequencing were carried out using standard PCR and sequencing techniques, largely following the protocol presented in Wahlberg & Wheat [30]. Sequencing was performed mainly with an ABI 3730 capillary sequencer.

### Phylogenetic analyses

The sequence alignments were done manually using BioEdit 7.0.9.0. There was very little variation in gene lengths among examined taxa, and therefore the sequence alignment could be done unambiguously through all taxa. However, a short region of the *wingless gene* was removed due to ambiguities in alignment. Similarly, a short region from the beginning of RpS5 was removed because of repetitive codons and resulting difficulties in alignment. We constructed neighbour-joining trees separately for each gene using Mega 4.0.1 and checked them carefully for identical sequences and otherwise doubtful patterns. Consequently, some contaminated taxa were re-analysed or removed from the subsequent analyses.

We made several trials with varying taxon and gene combinations. This was aimed at recognizing possible “rogue” taxa and further to search for potentially contaminated sequences. As a result, a taxon preliminarily identified as *Stamnodes* sp. was removed from the eventual analyses as being possibly contaminated or not actually being a member of this genus. We also examined the effects of exclusion of mitochondrial sequence data (analysis repeated three times), exclusion of gene partitioning (analysis repeated three times), as well as the effects of the third codon position (analysis performed once).

The phylogenetic analyses were carried out with model-based (Maximum Likelihood) methods. The eventual maximum likelihood analyses were carried out under the GTR+G model and the data were partitioned by genes. The maximum likelihood analysis was implemented using RAxML 7.2.6. [31] at the CIPRES Web portal [32]. Supports for nodes were evaluated with 1000 bootstrap replicates of the data. The eventual analysis was repeated 10 times to examine if rapid algorithms applied in RAxML consistently found the same global optimum and to map alternative positions of unstable taxa.

All trees were rooted to *Bombyx mori* Linnaeus (Bombycoidea, Bombycidae), which is certainly a non-geometroid taxon, but probably not a distant relative to Geometroidea among all Lepidoptera [29].

## Results

### Effects of varying taxon and gene combinations

The effects of varying taxon and gene combinations were compared against the analyses where all eight genes, third codon positions and data partition by genes were included. The removal of third codon position and partitioning by genes had little effect to the topology and node supports. A notable exception was the enigmatic *Ergavia roseivena* Prout, 1910. It always grouped within the Sterrhinae in partitioned analyses, while it never did so in non-partitioned analyses, where it was placed as sister to the Larentiinae (bootstrap support values ranging from 27–31 in ten replications). Omission of mitochondrial CO1 gene also had little effect to the tree topology, but weakened support values in most of the basal nodes.

Based on these trials, we decided to include all genes and third codon positions as well as partition the data by genes in the eventual analyses, as their inclusion obviously improved bootstrap support values between the closely related taxa, while not weakening the support values at the basal nodes. The ten repeats of the eventual data set yielded largely the same topology, with slight variation observed mostly in the apical nodes, suggesting that the independent heuristic searches recovered the same global optimum or at least ended close to it. The only major exception was the position of the Sterrhinae, which were found as sister group to the Larentiinae in six repeats, these subfamilies being together the sister group to all other Geometridae (support values ranging from 93–96). In four occasions the Sterrhinae were sister to all other Geometridae (support values ranging from 24–28).

### Major phylogenetic patterns

**Figure 1:**
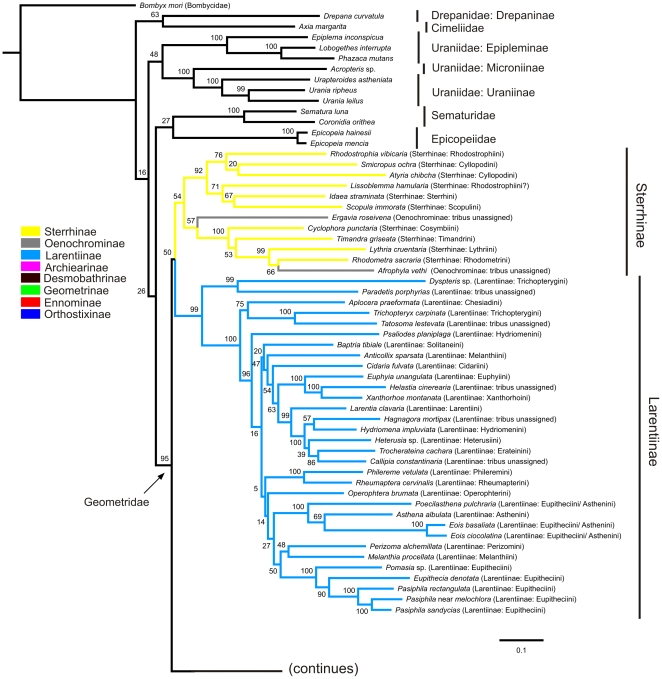
Overview of the 174 taxon RAxML maximum likelihood analysis. Tree shows the Geometridae subfamilies from Sterrhinae to Larentiinae. The tree was rooted on Bombyx mori Linnaeus, 1758. Non-Geometridae clades are shown in black, see Mutanen et al. [29]. Colours indicate subfamily associations and parentheses describe tribus associations of the analysed taxa prior to the analysis. Columns on the right indicate the subfamilies as they are recognized in this study.

**Figure 2:**
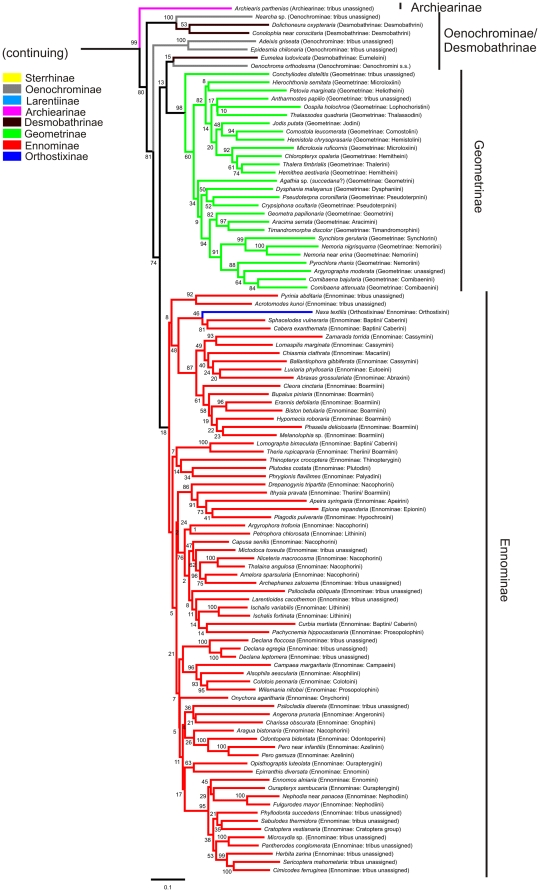
Overview of the 174 taxon RAxML maximum likelihood analysis. Tree shows the Geometridae subfamilies from Archiearinae to Ennominae. The tree was rooted on Bombyx mori Linnaeus, 1758. Colours indicate subfamily associations and parentheses describe tribus associations of the analysed taxa prior to the analysis. Columns on the right indicate the subfamilies as they are recognized in this study.

Our analysis of the phylogenetic relationships of the Geometridae resulted in a maximum likelihood tree with several clear patterns. The best obtained tree is shown in Figure 1 and Figure 2.

A clade consisting of the Epicopeiidae and Sematuridae is the sister-taxon to the Geometridae (bootstrap values ranging from 22–26 in ten replications). Uraniidae form a monophyletic group (46–57), being positioned between the above-mentioned clade and the Drepaniidae+Cimeliidae clade.

Monophyly of the Geometridae is well supported (93–96). According to our sampled species of the Geometridae, the Sterrhinae and Larentiinae were found to be sister to the remaining taxa, either Sterrhinae as the most basal (4/10) or Sterrhinae and Larentiinae as sister-taxa (6/10). Sterrhinae always came out as a monophyletic entity, with two branches, but the bootstrap support is low (47–59), as do the Larentiinae, but the branch is better supported (97–99). Structurally homogenous Archiearinae (98–100) were represented by only one species, appearing as sister to the Oenochrominae+Desmobathrinae complex and Geometrinae+Ennominae (73–86). Oenochrominae came out as non-monophyletic assemblage, with two species clustering in the Sterrhinae. Oenochrominae *sensu stricto* (5/10) or Oenochrominae *sensu stricto*+Desmobathrinae: Eumeleini (5/10) came out as the sister-group to the Geometrinae. The postulated sister-relationships were weakly supported. When Oenochrominae *sensu stricto* was placed alone as the sister-taxon to the Geometrinae, Eumeleini were grouped together with *Plutodes* in the Ennominae, next to a clade formed by *Pyrinia*+*Acrotomodes*. Monophyly of the Geometrinae is well supported (91–100). Within the Geometrinae, *Conchyliodes distelitis* Prout, 1930 is always positioned as sister to the remaining Geometrinae, which is divided into two branches (56–66). The monophyly of the Ennominae is only weakly supported (13–32). Orthostixini and Alsophilini, which have been considered subfamilies until recently, were both placed within the Ennominae.

In our analysis many tribes of Geometridae as hypothesized in traditional taxonomy were found to be non-monophyletic.

## Discussion

The trials we did to explore the effects of data partitioning as well as removal of third codon positions and mitochondrial data affected little the tree topology, and also had little effects to the node supports. There is no consensus whether or not relatively rapidly evolving mitochondrial sequence data should be included in studies that aim at resolving deeper-level phylogenetic patterns. Similarly, it is not clear if inclusion of third codon positions generally might blur rather than elucidate detecting phylogenetic patterns, as most changes in third codon positions do not involve changes in amino acids, being therefore selectively neutral and potentially increasing the amount of homoplasy. A likely explanation for the negligible effect of various data sets on tree topologies in our study is that most groupings are robust enough. Larentiinae and Sterrhinae were the only major taxa whose systematic positions remained somewhat unclear. Perhaps the only way to shed more light on those cases is the addition of data. Our results also suggest that mitochondrial CO1 gene provides additional data that is both informative and consistent with nuclear genomic data. While the third codon position of CO1 changes rapidly in time and probably contain little useful phylogenetic information, the first and second positions are stable enough to contain phylogenetically useful information at this phylogenetic level.

### Major phylogenetic patterns of the Geometridae and related taxa

Although we did not primarily aim at investigating the sister group of Geometridae, the results yielded some interesting patterns deserving attention. Our results on the sister-taxon to the Geometridae do not fully agree with the extensive analysis on the Ditrysian Lepidoptera by Mutanen *et al.* [29]. In their analysis, the clade consisting of Sematuridae+Uraniidae is monophyletic, being the sister-taxon to the Geometridae, whereas the Epicopeiidae were grouped together with the Lasiocampidae. The latter clade (Epicopeiidae+Lasiocampidae) was found to be the sister-taxon to the Geometroidea, although the supporting bootstrap values were low (22–26 in ten replications). Regier *et al.* [33] found Uraniidae to be the sister-taxon to the Geometridae and Epicopeiidae grouped together with the Sematuridae. In our analysis the Epicopeiidae and Sematuridae also grouped together, and we found these to be sister-taxa to the Geometridae, not nested within the Uraniidae. Other published molecular studies, e.g. [21], [23], [34], were not designed to analyse the sister-taxon question. Based on morphology, Minet [35] and Holloway [10] postulated the Geometroidea to consist of an unresolved trichotomic clade made up of the Geometridae, Sematuridae and Uraniidae. The Epicopeiidae were grouped as a sister-taxon to the Drepanidae, forming together the Drepanoidea.

Our analysis supports the result of Regier *et al.* [33] and Mutanen *et al.* [29], combining Cimeliidae with Drepanidae, and this clade is not closely related to the Geometridae. Uraniidae is a monophyletic entity with two separate lineages: Epipleminae+Microniinae and Uraniinae. Mutanen *et al.* [29] recovered the same pattern, whereas in Regier *et al.* [33], who only analysed one species from each subfamily, Uraniinae+Microniinae grouped together, and Epipleminae stood on its own.

### Major phylogenetic patterns of the Geometridae

Our extensive analysis on the phylogenetic relationships of the Geometridae, which generally agrees with the other recent studies, emphasizes that the general phylogenetic patterns and major lineages of the Geometridae are now well established. Of the geometrid subfamilies, the largest ones in terms of species count, *i.e.* Sterrhinae (with some previously misplaced taxa), Larentiinae, Geometrinae and Ennominae are consistently found as monophyletic lineages. Of these, the monophyly of the first three subfamilies is well supported, while support for the monophyly of Ennominae is generally lower. However, this large subfamily is regularly recovered as monophyletic in various trials, and we thus consider their monophyly to be relatively well supported as well. At the subfamily level, our results agree with those of Regier *et al.* [33], Wahlberg *et al.* [23], Mutanen *et al.* [29] and Yamamoto & Sota [21], except that the last reference maintained the Orthostixinae as valid at subfamily level, and grouped it together with the Desmobathrinae as a sister-taxon to the Archiearinae. In our analysis the Orthostixini grouped within the Ennominae. In both analyses the Orthostixini were represented by a *Naxa* species whose relationship to the type genus *Orthostixis* Hübner, 1823 still awaits detailed analysis. Regier *et al.* [33], Mutanen *et al.* [29] and Wahlberg *et al.* [23] did not include a representative of the Orthostixini.

Monophylies of both the Oenochrominae and Desmobathrinae are questioned. Holloway [8] revived the Desmobathrinae, to contain the delicately built ‘oenochromine’ genera with elongated, slender appendages. He failed to find unambiguous synapomorphies to unite the two included tribes, the Desmobathrini and Eumeleini– but each one of the two tribes can be defined on much stronger characteristics. In our analysis the two mentioned tribes did not group together. Scoble and Edwards [36] proposed a stricter definition of the Oenochrominae to apply to a group of robust-bodied Australian genera. Even with this stricter definition applied, the composition remained uncertain and they failed to find uniquely derived apomorphic characters. Their definition relied on general similarities of facies, wing venation and male genitalia structure. Cook and Scoble [6] suggested that the circular form of the lacinia and its orientation parallel to the tympanum in the tympanic bulla was an autapomorphy for these robust Oenochrominae. Holloway [8] noted that these features are not apparent in *Sarcinodes*, the only Oriental representative of the group. In our analysis *Oenochroma orthodesma* (Lower, 1894) was the sole representative of Oenchrominae *sensu sricto* in the sense of Scoble and Edwards [36].

### Lower-level interrelationships

#### Sterrhinae

Sterrhinae were found to have two major lineages, supporting the earlier morphology-based results [9], [13], and molecular-based results [24], [37]. Furthermore, the genus *Lythria* Hübner, 1823 (Lythriini) was placed in the Sterrhinae, grouping together with the Rhodometrini, agreeing with the recent finding [22] that *Lythria* is a genus in the subfamily Sterrhinae, not the Larentiinae. On a more detailed level, the Cosymbiini, Timandrini and Rhodometrini were found to be related in the same sequence in the present study as in Sihvonen and Kaila [13], Õunap [37] and Strutzenberger *et al.* [24]. Holloway [9] treated these three tribes as an unresolved trichotomy. In the present analysis the Rhodostrophiini and Cyllopodini grouped together, as did the Sterrhini and Scopulini. The same pattern was proposed by Holloway [9], Sihvonen and Kaila [13], Õunap [37] and Strutzenberger *et al.* [24], although the latter two did not have Cyllopodini included in the analysis. The systematic position of *Lissoblemma* Warren, 1902 and related genera, see [13], remains problematic. These genera have certain structural features that suggest a relationship with either Rhodostrophiini or Scopulini. In the present analysis *L. hamularia* (Snellen, 1872) grouped next to the Sterrhini in the Scopulini+Sterrhini lineage. *Afrophyla vethi* (Snellen, 1886) and *Ergavia roseivena* Prout, 1910, which had earlier been included in the Oenochrominae, were found to be associated with the Sterrhinae. However, the Sterrhinae association of *E. roseivena* was not found in non-partitioned data analysis, where it was placed as sister to the Larentiinae, so this association remains somewhat doubtful. More extensive studies are needed to resolve their exact position.

#### Larentiinae

The tribe Trichopterygini, which is diagnosed by the male hindwing anal area being reduced and modified into a lobe, has been proposed to be the sister to the remaining Larentiinae [9]. In our analysis this view did not gain support because the genera *Trichopteryx* Hübner, 1816 and *Tatosoma* Butler, 1874 grouped together, being sister to the genus *Aplocera* Stephens, 1827, which is currently placed in the Chesiadini [38]. The sister position to the remaining Larentiinae was occupied by the Neotropical *Dyspteris* sp. and *Paradetis porphyrias* (Meyrick, 1883) from New Zealand that grouped strongly together (bootstrap values ranging from 98–100 in ten replications). Strutzenberger *et al.* [24] also found *Dyspteris* to be the first branching taxon in the Larentiinae. The potential association of these two taxa to the true Trichopterygini requires further investigation. Hodges *et al.* [39] placed *Dyspteris* in the Lobophorini, which Holloway [9] included in the Trichopterygini.

The genus *Baptria* Hübner, 1825 has been placed in the Solitaneini (*vide* [38], [40]), which in turn has been proposed to be a junior synonym of Operophterini [25]. If the tribal synonymy holds, then our analysis suggests that *Baptria* does not belong to the Solitaneini. Our results also question the association of the genus *Anticollix* Prout, 1938, with the Melanthiini as it did not group together with the type genus of the tribe, *Melanthia* Duponchel, 1829. There are two large groupings within the Larentiinae, but the split is weakly supported (bootstrap values ranging from 3–30 in ten replications). The first group includes genera from *Baptria* to *Callipia* Guenée, 1858, representing many Holarctic tribes, but also taxa from New Zealand, several species from South America, many of which have not been assigned to a tribe. The three genera, *Helastia* Guenée, 1868, *Hagnagora* Druce, 1885, and *Callipia* Guenée, 1858, which have not been assigned to currently valid tribes, all fall within the first group. The New Zealand genus *Helastia*, diagnosed and illustrated in Craw [41], groups together with the Xanthorhoini (99–100). Association is also supported by the similar facies and structures of the genitalia (see also [9]). The South American genera *Hagnagora* and *Callipia* grouped together with the Hydriomenini, Heterusiini and Erateinini tribes (96–99). The second group includes genera from *Philereme* Hübner, 1825 to *Pasiphila* Meyrick, 1883.

Holloway [9] and Holloway *et al.* [42] have noted the Eupitheciini, Operophterini and Perizomini to share an unusual set of structures associated with the juxta of the male genitalia, the dorsal ones are termed labides with the ventral ones extending down towards each side of a central constriction of the juxta. The labides on each side are independent or only partially united in the Eupitheciini but fully fused in the Operophterini and Perizomini. The tribe Asthenini may also be related [9], though the structures Pierce [43] refers to as labides are not entirely similar to those in the other three tribes. Holloway [9] placed *Poecilasthena* Warren, 1894 and *Eois* Hübner, 1818 in the Eupitheciini, but indicated that these genera could be placed in the Asthenini, as is done in McQuillan and Edwards [44]. Our results give support to the hypothesis that the tribes discussed are closely related, particularly the Eupitheciini and Perizomini, the latter being associated with the Melanthiini. As far as we know, the potential Melanthiini+Perizomini relationship has not been discussed earlier. Melanthiini have been subordinated to Rheumapterini [45], and based on characters of the pupa a Hydriomenini relationship has also been postulated [46].

The Asthenini seem to form a monophyletic lineage, sister-taxon to the above-mentioned three tribes. Our results support the placement of *Poecilasthena* in the Asthenini, agreeing with [44], [47] and placement of *Eois* in the Asthenini, tentatively proposed by Holloway [9]. The view of Xue and Scoble [47], who excluded *Eois* from the Asthenini, is not supported. Their arguments were based on the absence of the labides and the presence of distinctive signum that in their view differ markedly from that seen in typical Asthenini. Strutzenberger *et al.* [24] did not have the Asthenini in their analysis of *Eois*; Phileremini was found to be in a sister-position to the genus *Eois*.

#### Archiearinae

The Archiearinae are a small group of diurnal moths, with a strikingly disjunct distribution, and traditionally presumed to be the most basal group of the Geometridae, e. g. [9], [15], [48]. In the recent molecular analyses, which included considerably fewer taxa than the present one, the Holarctic Archiearinae have been placed as basal to the Geometrinae+Ennominae [20], [23], [34]. Yamamoto & Sota [21] found Archiearinae to be the sister-taxon to the Orthostixinae+Desmobathrinae clade, which in turn was sister to the Geometrinae+Ennominae clade. Our results agree with those mentioned above, the Archiearinae being a sister-taxon to the Oenochrominae+Desmobathrinae complex and Geometrinae+Ennominae. The Chilean genus *Archiearides* Fletcher, 1953 [49] has been shown to be a sister-taxon to the Holarctic *Archiearis* Hübner, 1823, whereas the Tasmanian ‘Archiearinae’ have been shown to be misplaced in the Archiearinae and have close affinities to the Australian Nacophorini (Ennominae) [20]. These findings may suggest that the lack of an accessory tympanum in the Archiearinae is a secondary adaptation to a diurnal habit and not a primitive character [20].

#### Oenochrominae+Desmobathrinae complex

The ‘Oenochrominae’ have, from the very beginning, been conceived as a polyphyletic assemblage of groups not fitting the venation characteristics of the other subfamilies. Over a long period, the concept included the geometrid subfamilies Alsophilinae, Desmobathrinae and Orthostixinae, until Scoble and Edwards [36] proposed that a stricter definition of Oenochrominae should be applied to robust-bodied Australian genera. Despite this, unique, diagnostic morphological characters have been difficult to find.

The Desmobathrinae are a pantropical group revived by Holloway [8] to contain the delicately built ‘Oenochrominae’ genera that have elongated, slender appendages (legs, antennae). The two recognized tribes, Desmobathrini and Eumeleini, are diagnostic, but attempts to find clearly established synapomorphies to unite them have failed [8].

In our results the ‘Desmobathrinae’ formed a grade rather than a clade, and the single true oenochromine in our study was often associated with *Eumelea* Duncan & Westwood, 1841 (Desmobathrinae), though with low support value. Such an arrangement would be plausible on morphological basis as well, as no synapomorphies are known to unite the Desmobathrinae *sensu* Holloway [8]. Because of the limited taxon sampling in this subfamily, the number of separate lineages may increase considerably with a better coverage. The *Nearcha* Guest, 1887 grouped together with two Desmobathrini taxa, the clade has high support values (bootstrap values ranging from 98–100 in ten replications), and the three represented taxa appear to have a Gondwanan type distribution (*Nearcha*: Australia, *Dolichoneura* Warren, 1894: South America, *Conolophia* Warren, 1894: South Africa). The position of Eumeleini is problematic: in 5 replications it grouped together with Oenochromini *sensu stricto* (13–22), but in 5 other replications it grouped together with *Plutodes costatus* Butler (Butler, 1886) (Plutodini) in the Ennominae (27–35). The Eumeleini have a number of unusual features—in the male genitalia the uncus is cruciform and the tegumen is distinctly shaped [50]—setting it apart from the Desmobathrini and most other Geometridae [8]. These structures are not found in *Plutodes* Guenée, 1858 either. Based on morphology, Beljaev [51] placed the Eumeleini in the Geometrinae.

Holloway [8] has suggested that the Desmobathrinae might represent the sister-group to the Geometrinae as *Celerena* Walker (not included in our analysis) has high concentrations of geoverdin, the pigment that characterizes the geometrines [52]. In our analysis the Desmobathrinae: Eumeleini+Oenochromini *sensu stricto* (5/10 replications) or Oenochromini *sensu stricto* (5/10) alone stood as a sister-group to the Geometrinae. The Eumeleini did not yield high concentrations of geoverdin [52].

#### Geometrinae

Traditionally the Geometrinae have been classified to contain the Dysphaniini, with the remaining taxa being classified into as many as seventeen tribes, summarized in Forum Herbulot [25]. The Dysphaniini have high geoverdin concentrations, sharing this and a few morphological features with the Geometrinae, suggesting these taxa may be linked. The lack of shared, unique characters has led some authors to challenge the placement of the Dysphaniini in the Geometrinae [6], [8], [52]. The remaining geometrine tribes are difficult to diagnose, and some genera do not fall readily into any of them, *vide* e.g. [53]. This lead Holloway [8] to suggest that all non-Dysphaniini taxa should be joined as one large tribe, the Geometrini, with the tribes recognized by other authors, e.g. [53], [54] classified as subtribes of the Geometrini.

Our results indicate two major groupings in the Geometrinae (bootstrap values ranging from 56–66 in ten replications), but we did not find support for the division between the Dysphaniini and the remaining Geometrinae. The Dysphaniini is clearly associated with the Geometrinae, grouping together with the Pseudoterpnini. Therefore our results do not lend support to the ‘Dysphaniini – Geometrini’ hypothesis presented by Holloway [8], but it must be noted that bootstrap values of most nodes are really low. We included three geometrine taxa from Africa in the analysis, whose systematic position had remained uncertain. *Antharmostes* Warren, 1899 grouped together with the Lophochoristini+Thalassodini clade, *Argyrographa* Prout, 1912 grouped together with the Comibaenini, and peculiar, monotypic *Conchyliodes* Prout stood on its own, being the sister-taxon to the rest of the Geometrinae. *Conchyliodes distelitis* lacks any green colour; the wings are uniform white with distinct brown margins that are mixed with red scales, particularly on the underside. Prout [55] included the genus in the Geometrinae (Hemitheinae) based on its venation, noting that its genitalia shows no affinity to any of the other Geometrinae. The subfamily association has been adopted in later works, e.g. [56], [57], and is supported by our data.

#### Ennominae

The subfamily Ennominae is diagnosed primarily by the loss of vein M2 in the hindwing, or more precisely, the vein is reduced to a fold rather than being expressed as a tubular vein. There are apparent reversals of this in a few genera, e.g. the New World genera *Anavinemina* Rindge, 1964 and *Melanolophia* Hulst, 1896 [11] and the Holarctic *Epirranthis* Hübner, 1823. Our analysis also confirmed the placement of several taxa, which have a tubular hindwing vein M2 present, in the Ennominae. These include the Alsophilini (also in [21], [34]), the Orthostixinae/Orthostixini and potentially also the Eumeleini. For Eumeleini, see discussion above. Alsophilini were grouped with the Colotoini as in Wahlberg *et al.* [23].

In Yamamoto & Sota [21], and in our analysis, the Orthostixinae/Orthostixini were represented by a *Naxa* Walker, 1856 species. *Naxa seriaria* (Motschulsky, 1866) from Japan grouped together with the Desmobathrinae and Archiearinae clade [21], whereas in the present analysis *N. textilis* Walker, 1856 from Taiwan grouped together with the Baptini/Caberini clade in the Ennominae. We are unable to speculate what may have caused the different positioning of *Naxa*, except that in our analysis different markers and different outgroup were used, and that our analysis was more extensive, both in number of species and genes analysed. Holloway [8], who also examined the type genus of the tribe, *Orthostixis* Hübner, 1823, suggested that the Orthostixini may possibly be an Ennominae, thus agreeing with our result. Holloway based his view on a comparison of numerous morphological features from the adult, larva and pupa. Later Holloway [9] treated the Orthostixinae as valid at subfamily level, placing it as sister-group to the rest of the Ennominae.

Overall, the bootstrap values taken for many sub-lineages within the Ennominae are rather high, but often the interrelationships are weakly or very weakly supported. For this reason we will discuss the interrelationships rather superficially.

The Cassymini, Eutoeini, Macariini and Boarmiini in the broad sense have been proposed to form a monophyletic group, sharing reduction of the hooklets of the pupal cremaster to a strong terminal pair and development of a fovea of various forms in the male forewing [7], [42]. Our results support this grouping, including the Boarmiini *sensu* Holloway (bootstrap values ranging from 83–92 in ten replications), and are in general agreement with those of Wahlberg *et al.* [23]. The only refinements compared to Holloway [7] are the inclusion of the Abraxini in the same clade, and the exclusion of *Charissa obscurata* (Denis & Schiffermüller, 1775) and the Theriini. The latter does not fall within the broad concept of the Boarmiini, but it is associated with the genus *Lomographa* Hübner, 1825 (Baptini/Caberini), a result also found by Wahlberg *et al.* [23]. Our data suggests separating the Baptini (with genus *Lomographa* Hübner, 1825) from the Caberini. Viidalepp [58] combined *Ithysia pravata* (Hübner, 1813) with the Theriini, but in our analysis it grouped together with the clade containing representatives of the tribes Apeirini, Epionini and Hypochrosini and an African representative of the genus *Drepanogynis* Guenée, 1858, the last taxon expected to fall in the Nacophorini [59].

The genera *Acrotomodes* Warren, 1895 and *Pyrinia* Hübner, 1818 always grouped together but their position was unstable. They were placed as the most basal Ennominae with very weak support (8/10 replications, bootstrap values ranging from 3–30) or with the Azelini (2/10 replications, bootstrap values ranging from 11–12). Pitkin [11] grouped these two genera together, but did not assign them into a tribe, noting that they have some apomorphic features in common with *Falculopsis* Dognin, 1913 (not included in our analysis). In these three genera the valva is divided, and the chaetosemata extend across the back of the head, a feature in common with the Macariini [11], [12].

The Nacophorini appeared polyphyletic, somewhat intermingled with the Lithinini. *Drepanogynis tripartita* (Prout, 1915) and monotypic *Aragua* Rindge, 1983 should perhaps be removed from the Nacophorini, whereas *Mictodoca* Meyrick, 1892 and *Archephanes* Turner, 1926 from Australia should perhaps be included. The position of the New World Nacophorini, here represented by the monotypic genus *Aragua* Rindge, 1983 must be investigated in more detail. Our results suggest that it is unrelated to Australian and African Nacophorini. Broader taxon sampling in this species-rich lineage is required.

The African genus *Psilocladia* Warren, 1898 (tribe unassigned) was represented in the analysis by two species; the type species of the genus *P. obliquata* Warren, 1898 and *P. diaereta* Prout, 1923. These did not group together, suggesting that *P. diaereta* may be misplaced in *Psilocladia*. Results from DNA barcoding of the CO1 fragment (BOLD database) show a close similarity between *P. diaereta* and *Xenimpia* Warren, 1895, which in all likelihood may be closely related.

The monotypic African genus *Larentioides* Prout, 1917 has not been assigned to currently valid tribes. In our analyses its placement remained ambiguous because the association with the *Psilocladia*, *Ischalis* Walker, 1862, *Curbia* Warren, 1894, and *Pachycnemia* Stephens, 1829 group of genera was weakly supported (bootsrap values ranging from 7–80). The previously unassigned New Zealand genus *Declana* Walker, 1858 grouped in all ten replications as sister to the clade containing the Alsophilini, Colotoini, Prosopolophini and Campaeini, but the support was weak (8–21).

The Neotropical Palyadini, which are diagnosed by the lack of a frenulum and retinaculum wing-coupling system [60], grouped weakly together with the Plutodini (5/10 replications, bootstrap values ranging from 34–41) or when the Eumeleini grouped together with the Plutodini, the Palyadini grouped together with the Baptini (5/10 replications, bootstrap values ranging from 14–25). Our results do not therefore shed much light on the difficult positioning of these moths. Hodges *et al.* [39] subordinated the Palyadini to the Baptini, Scoble [60] considered the original Guenée's concept of this group valid if the genus *Eumelea* (Eumeleini) is excluded, and Holloway remarked that there are morphological similarities between the Baptini and Palyadini. Pitkin [11] treated the Palyadini as a subgroup of the Caberini/Baptini.

The Odontoperini and Azelinini association is strongly supported (bootstrap values ranging from 99–100 in ten replications), already noted by Holloway [9] on morphological grounds. Beljaev [61] proposed the Azelinini to be related to the Ennomini *sensu lato* (*vide* [51]) on the grounds of male genitalic muscles. The systematic position of *Epirranthis diversata* (Denis & Schiffermüller, 1775) has remained controversial, being placed either in the Ennominae: Ennomini (e.g. [38], [58]), in the Oenochrominae *sensu lato* (e.g. [62], [63]) or in the Desmobathrinae [15]. In our analysis *E. diversata* grouped together with *Opisthograptis luteolata* (Linnaeus, 1758), the latter having been recently placed in the Ennominae tribe Ourapterygini, a position which is not well supported by our results.

The Ennomini and related taxa (Ourapterygini, Nephodiini, *Cratoptera* group and seven unassigned taxa) constitute a well-supported clade (bootstrap values ranging from 92–97 in ten replications). The Nephodiini and Ourapterygini group together; this close relationship, or even synonymy, has already been suggested by morphology [11]. The most apical Ennominae include the *Cratoptera* group and seven other Neotropical genera, with a few Nearctic species, which have not been assigned to a tribe [11]. The only exception is genus *Phyllodonta* Warren, which was placed earlier in the Ourapterygini [18]. Interestingly, the nine Neotropical ennomine taxa not previously assigned to tribal level only clustered together in two clades, the *Cratoptera* group and *Acrotomes*+*Pyrinia* clade, and were not phylogenetically scattered across the whole subfamily. Perhaps most of the remaining 56 genera not assigned to a tribal level by Pitkin [11], may also group in one of these two clades.

### Future

In the future the few remaining geometrid taxa, which were not included in this analysis, should also be analysed in a broader context. These most notably include the African Diptychini, which is potentially a distinct lineage of the Ennominae, perhaps related to the Ourapterygini [8]. Prout [55] placed some of the genera in the Oenochrominae, whereas Janse [56] was the first to diagnose this group of four genera as Diptychini (Diptychinae in the modern sense), considering them as potentially related to the Larentiinae. Pinhey [64], Staude [65] and Vári *et al.* [66], following the general elevation of the tribes to subfamily level, treated the Diptychinae as a subfamily. Staude [65] reviewed the taxonomic history of the group adding two more genera. We tried to analyse three different Diptychini taxa, but all attempts were unsuccessful. Inclusion of the genus *Orthostixis*, type genus of the Orthostixini, in a DNA analysis is important because relationships of the *Orthostixis* and the genera used as a surrogate are somewhat tentative. The other missing tribes, indicated in Table S1, may be somewhat trivial from the phylogenetic point of view. Many of them were diagnosed by Holloway [7], [8]. We also suggest denser taxon sampling and further analyses to more accurately resolve the Oenochrominae+Desmobathrinae relationships.

Many geometrid genera are still unassigned to tribe, at least partly because the current classification is strongly biased towards European fauna, whereas the vast majority of geometrid diversity resides in the tropics. Future phylogenetic analyses should vigourously try to take this mismatch into consideration.

## Supporting Information

Table S1 **Taxonomic details for sequenced taxa.** The list contains all valid tribal names provided in the *Forum Herbulot world list of family group names in the Geometridae* [25], based on Holloway [7]–[10], supplemented from Beljaev [26]. References [1]–[91] indicate the recent literature, where tribus association has been used.
(XLS)

Table S2 **Sequencing success in studied taxa.** GenBank accession numbers provided for successfully amplified genes, “missing” indicates lack of sequence data.
(XLS)
